# Comparison between the 6-minute walk tests performed in patients with chronic obstructive pulmonary disease at different altitudes

**DOI:** 10.1590/S1679-45082014AO3139

**Published:** 2014

**Authors:** Selma Denis Squassoni, Nadine Cristina Machado, Mônica Silveira Lapa, Priscila Kessar Cordoni, Luciene Costa Bortolassi, Juliana Nascimento de Oliveira, Cecilia Melo Rosa Tavares, Elie Fiss

**Affiliations:** 1 Faculdade de Medicina do ABC, Santo André, SP, Brazil.

**Keywords:** Pulmonary disease, chronic obstructive/metabolism, Exercise test/methods, Exercise tolerance, Walking, Altitude

## Abstract

**Objective:**

To evaluate the influence of the altitude on the 6-minute walking test in patients with moderate to severe pulmonary disease.

**Methods:**

Twenty-nine patients performed the 6-minute walk test at a pulmonary rehabilitation clinic in Santo André (above sea level), in São Paulo State, and at the Enseada Beach, in Guarujá (at sea level), also in São Paulo State. Of these 29 patients, 8 did the test both on hard sand and on asphalt to analyze if there were differences in performance during the tests. Data such as heart rate, oxygen saturation, test distance, and Borg scale were compared.

**Results:**

We found no statistical difference in relation to oxygen saturation at rest before the beginning of the walking test in Santo André 94.67±2.26% and at sea level 95.56±2% (p=0.71). The minimum saturation measured during the test was 87.27±6.54% in Santo André and 89.10±5.41% in Guarujá (p=0.098). There were no differences in the performed distance between the different kinds of terrains; the distance on sand was 387.75±5.02m and on asphalt it was 375.00±6.54m (p=0.654). Regarding oxygen saturation during walking, the pulse oximetry on sand was 95.12±1.80% and on asphalt it was 96.87±1.64% (p=1.05).

**Conclusion:**

Altitude did not affect the performance of the walking test in patients with moderate to severe pulmonary disease and the results were similar in both cases, on sand and on asphalt.

## INTRODUCTION

Individuals with chronic obstructive pulmonary disease (CPOD) may present with a significant reduction in physical capacity due to various factors, such as dynamic hyperinflation and progressive physical deconditioning associated with inactivity,^(1)^ leading to physical and social limitations, which cause deterioration in their quality of life.^(2)^

Resulting from these alterations, evaluation of physical capacity or of the capacity for exercise in individuals with CPOD became the goal of many studies. The causes of physical exercise intolerance in patients with CPOD are traditionally focused on the limitations of the ventilatory system (gas exchange),^(3,4)^ in functional abnormalities (such as reduced strength and resistance), and in muscular bioenergy (such as reduced oxygen uptake).^(5-7)^

In order to evaluate physical capacity of patients with CPOD, the 6-minute walk test (6MWT) is used, which more accurately reflects the limitations to Daily Life Activities and is more sensitive than treadmill tests.^(8)^ Individuals with CPOD present with reduced tolerance to exercise,^(9,10)^ associated with a feeling of dyspnea and fatigue, and suffer reduction of functional capacity upon exercise with the progression of the disease.^(9 ) ^Studies performed at the Dead Sea showed that patients with CPOD showed an increase in the distance covered during the 6MWT, with less dyspnea and improved functional capacity at lower altitudes.^(11-16)^

The effects of altitude on pulmonary physiology are a result, basically, of decreased atmospheric pressure. As the altitude we are exposed to increases, there is a decrease in atmospheric pressure and consequently, a drop in the partial pressure of oxygen in the environment.^(17 ) ^Nevertheless, the interference in the variation of 6MWT at different altitudes is still not well established, such as, for example, between the city of Santo André (São Paulo) and at sea level.

The initial hypothesis of the study was that the patients with moderate to severe chronic obstructive pulmonary disease would have a better performance at sea level during the 6-minute walking test, regardless of the type of soil.

## OBJECTIVES

To evaluate the influence of altitude on the 6-minute walk test in patients with chronic obstructive pulmonary disease, moderate to severe, and the performance of the patients on different soils, such as hard sand and asphalt.

## METHODS

This is a retrospective study in which 29 patients were analyzed, 19 of them women. The mean age of patients was 66.41±10.55 years, with a forced expiratory volume in the first second (FEV1), in percentage, of 50.56±14.81%, and body mass index (BMI) of 25.9±5.9. The subjects had moderate to severe CPOD, according to GOLD,^(18)^ in which 17 were moderate and 12 were severe cases (Table 1).

**Table 1:** Demographic characteristics of the sample

Characteristics	n
Gender	
Male	10
Female	19
Age, years	
Mean	66.41
Standard deviation	14.81
BMI, m/kg^2^	
Mean	25.9
Standard deviation	5.9
Post-baseline FEV1	
Mean	50.56
Standard deviation	14.81
CPOD	
Moderate	17
Severe	12

BMI: body mass index; FEV1: forced expiratory volume in the first second; CPOD: chronic obstructive pulmonary disease.

The patients carried out the 6MWT at the Pulmonary Rehabilitation Outpatient Clinic of the *Faculdade de Medicina do ABC*, in the city of Santo André (above sea level - 760m), in São Paulo, and on the Enseada beach, in Guarujá (at sea level=0m), which is also in the state of São Paulo. The test was done as per the guidelines of the American Thoracic Society (ATS)^(19)^ on a 30-meter marked lane. The 6MWT was done during an external activity, which was included in the Pulmonary Rehabilitation Program (PRP) as standard procedure of the facility during the period from October, 2012, to April, 2014. The project was approved by the Research Ethics Committee of the *Faculdade de Medicina do ABC* (CAAE No.: 06661712.3.0000.0082), and all patients signed the Informed Consent Form.

The study included patients with moderate to severe CPOD, with FEV1 between 30% and <80%,^(18)^ who were clinically stable and under adequate treatment for the degree of illness and under follow-up with a pneumologist. Excluded were clinically unstable patients who had had exacerbations within the previous 3 months, incapable of walking (musculoskeletal or neurological limitations) and with severe cardiac disease and/or requiring the use of oxygen.

Patients performed the 6MWT one hour after arriving at the beach, as per the ATS guidelines,^(19)^ with no adaptation period. Before and after the tests, parameters such as systolic (SBP) and diastolic (DBP) blood pressure, heart rate (HR), oxygen saturation (SaO_2_), and Borg scale^(20)^ for dyspnea and lower limb fatigue were checked. However, for purposes of safety and better monitoring of the subjects, SaO_2_, HR, and Borg’s scale were measured every minute until the end of the test. After completing the 6MWT, the distance that each individual had covered during the test was recorded. All the parameters and distances were compared between the tests done above sea level and at sea level. To check SaO_2_ and HR, a digital finger Onyx^®^, model 9500 (Nonin) pulse oximeter was used (Medical, Inc. Minneapolis, MN, USA). During this activity, the patients were followed by pneumologists, physical therapists, and a licensed professional nurse (LPN) trained for emergency measures.

A total of 29 patients participated in the study. Among them, eight patients did the test on the beach with an irregular surface (hard sand) and on asphalt on two different days, with the purpose of evaluating their performance on different terrains.

The data obtained from the total sample (n=29) during the tests, such as SaO_2_ and the distance of the 6MWT, were compared and correlated. The paired t-test and correlation between them and all the variables performed above sea level, at sea level, and on different surfaces of the beach (distance, SaO_2_, and Borg’s scale) were analyzed. For analysis of the Borg scale, maximum dyspnea, and lower limb fatigue**,** in Santo André and on the beach, the χ^2^ test was used. The only variables analyzed in the eight patients who did the tests on the sand and on the asphalt were the 6MWT distance and SaO_2_ during the tests, since the sample was small and χ^2^ test could not be done. The results were obtained with means and standard deviations. The sample normality test (even knowing that it should be done with larger samples) was performed and we chose to use the paired *t-*test, since the same sample was compared in different situations. The calculation of *r *was done by Pearson’s test.

## RESULTS

No differences were found between the SaO_2_ at rest during the 6MWT at the Outpatient Clinic in Santo André (94.76±2.26%) and at the Enseada beach (95.54±2%; p=0.71). The minimum SaO_2_ found during the tests was 87.28±6.54%, in Santo André, and 89.10±5.41%, in Guarujá (p=0.098) (Table 2).

**Table 2:** Mean variables of the 6-minute walk test at different altitudes

Altitude	Distance 6MWT, m			Baseline SaO_2_,%			SaO_2_ min,%		

	Mean	Standard deviation	p value	Mean	Standard deviation	p value	Mean	Standard deviation	p value
Above sea level=760m	428.31	54.94	0.178	94.76	2.22	0.71	87.28	6.43	0.098
At sea level=0 m	413.17	59.78		95.54	1.97		89.10	5.32	

SaO_2_: oxygen saturation; 6MWT: 6-minute walk test.

**Figure 1:**
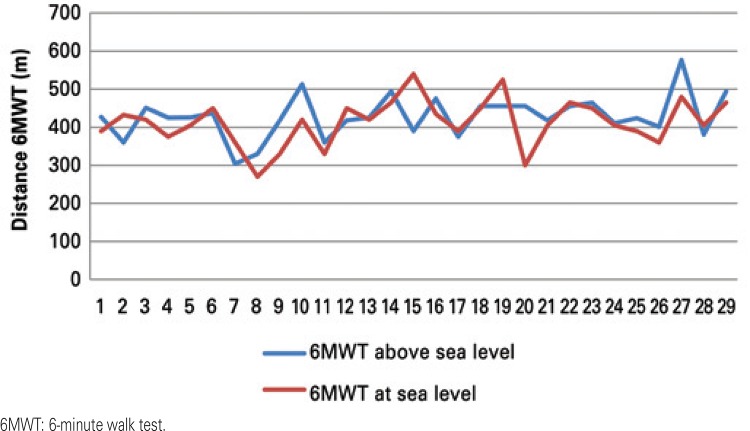
Comparison of the distance covered in the 6-minute walk test at different altitudes (n=29)

There was no statistical difference in the distances during the 6MWT performed on the beach and at the altitude in Santo André. On the beach, there was a mean of 428.31±55.91m, and in Santo André, 413.17±60.8m (p=0.178), that is, the patients were capable of reproducing the test at different altitudes (r=0.49; p=0.007) (Table 2) (Figure 1).

**Figure 2:**
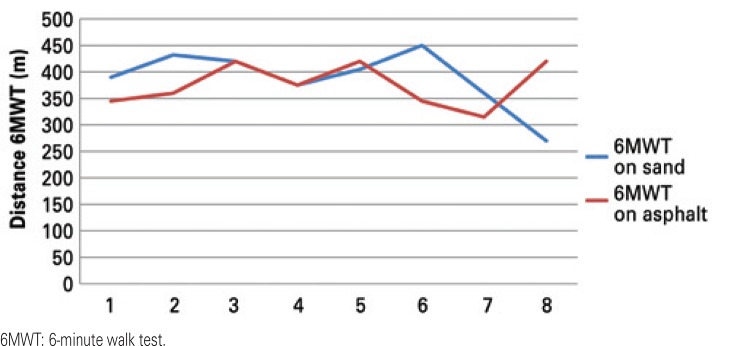
Comparison of the distance covered in the 6-minute walk test on different surfaces (n=8)

A sample of eight patients performed the 6MWT on the beach and on the asphalt and there was no difference as to the distance covered between the types of terrain (distance of 387.75±5.02m on the sand and 375.00±40.88m on the asphalt; p=0.654). During the walk on the sand, the SaO_2_ was 95.54±1.97% and on asphalt, 96.88±1.54% (p=1.05), that is, the test is reproducible on both surfaces (Table 3) (Figure 2).

**Table 3:** Mean variables of the 6-minute walk test on different types of soil at the beach

Types of soil	Distance 6MWT (m)			Baseline SaO_**2**_ (%)		
	Mean	Standard deviation	p value	Mean	Standard deviation	p value
Asphalt	375	38.24	0.654	96.88	1.54	1.05
Beach sand	387.75	5.02		95.54	1.97	


SaO_2_: oxygen saturation; 6MWT: 6-minute walk test.

For the maximum Borg value of dyspnea, there were no differences in χ^2^ test between the two situations (p=0.3), just as we found no significant difference relative to the maximum Borg value for lower limb fatigue (p=0.55), that is, the feeling of dyspnea and lower limb fatigue was similar at the different altitudes (Table 4).

**Table 4:** Data from the Borg scale(20) - dyspnea and fatigue of lower limbs

Borg Scale	Santo André	Beach	Asphalt
	(n=29)	(n=29)	(n=8)
Borg maximum dyspnea	7	7	5
Borg maximum fatigue of lower limbs	5	5	5
Borg minimum dyspnea	0	0	0
Borg minimum fatigue of lower limbs	0	0	0

## DISCUSSION

Patients with CPOD usually report that at sea level, the feeling of dyspnea is less than in the city of Santo André. The tests at both altitudes were reproducible, that is, they had the same performance both at sea level and at 760m altitude (similar results), regardless of the ground used.

The patients showed no difference in the distance between the 6MWT at the different altitudes evaluated or for the different types of surfaces, but a study that evaluated energetic and mechanical output of individuals who run and walk in the sand, demonstrated that walking and running in the sand requires 1.6-2.5 times more effort than on a firm surface.^(21)^ Whereas a study performed at the Dead Sea in patients with CPOD showed an increase in distance of the 6MWT from 112m after remaining for a week at an altitude of 417m below sea level, and of 170m after three weeks. One week after returning to Jerusalem, they continued with an increase in distance covered of 116m. In our study, we did not obtain this difference, but we should consider that the time that these patients remained at sea level was too short for there to be some alteration in 6MWT.^(12 ) ^Additionally, the difference in altitude was smaller (in meters) than in the study mentioned. The 6MWT is an important test for evaluating the physical capacity of individuals with functional limitation, in whom the assessment should be useful for quantifying the severity of this limitation and the results of treatment. The individuals with CPOD presented with reduced tolerance to exercise,^(9)^ associated with feeling dyspnea and fatigue, and suffer a reduction in functional capacity for exercise with the progression of the disease.^(10)^

We found no differences between the SaO_2_ at rest during the walking tests and at minimal saturation between the different altitudes. On the other hand, in the study performed at the Dead Sea, in Jerusalem, the patients presented with a decrease from 88 to 84% in SaO_2 _at the end of the walk. At the Dead Sea, this decrease was from 92 to 86% after one week, and from 93 to 83% after three weeks.^(12)^ Also, another study^(13)^ carried out at the Dead Sea, suggested that all the patients felt less dyspnea and reported improved functional capacity and lower need for oxygen. It is possible that the time of stay at lower altitudes influenced the results obtained and it may be necessary to remain for at least one week at lower altitudes to evaluate the differences in physical capacity after this period of adaptation.

Patients with CPOD presented with difficulty in socialization and leisure, did not accompany family members to social events, that is, they isolated themselves and quit participating in performing tasks and abandoning their activities.^(22)^ It is important to realize that when a person is diagnosed with CPOD, he/she should necessarily modify his/her lifestyle, seeking better quality of life.^(23)^ To stimulate social contact, as well as to promote the outing of these patients at the beach, is part of our PRP, with the objective of improving their quality of life and their integration with society.

Some limitations might have influenced the results of the present study, such as the short time of permanence at sea level, the greater distance between the altitudes studied, and the small size of the sample. As an objective of a future project, it would be interesting to follow this line of research and study patients who remain at least one week at sea level.

## CONCLUSION

The altitude did not influence the performance of the patients with moderate to severe chronic obstructive pulmonary disease during the 6-minute walk test. The results of the variables analyzed were similar, both at sea level and at the altitude of the city of Santo André (760m).

The analysis of the performance related to the difference surfaces also showed no significant difference in the 6-minute walk test performed on the asphalt or on hard sand, and it would be necessary to carry it out with a larger sample.
