# Supplementary material for: Comparison between the 6-minute walk tests performed in patients with chronic obstructive pulmonary disease at different altitudes
Source: Einstein (Sao Paulo). 2014 Oct-Dec;12(4):447–51. doi: 10.1590/S1679-45082014AO3139 (PMC4879910; doi:10.1590/S1679-45082014AO3139)
Supplement: Translation

# Translation: Comparação entre os testes de caminhada de 6 minutos realizados em pacientes com doença pulmonar obstrutiva crônica em diferentes altitudes

**Authors:** Selma Denis Squassoni, Nadine Cristina Machado, Mônica Silveira Lapa, Priscila Kessar Cordoni, Luciene Costa Bortolassi, Juliana Nascimento de Oliveira, Cecilia Melo Rosa Tavares, Elie Fiss

**Affiliations:** 1 Faculdade de Medicina do ABC, Santo André, SP, Brasil.

**Keywords:** Doença pulmonar obstrutiva crônica/metabolismo, Teste de esforço/métodos, Tolerância ao exercício/métodos, Caminhada, Altitude

## Abstract

**Objetivo:**

Avaliar a influência da altitude no teste de caminhada de 6 minutos em pacientes com doença pulmonar moderada a grave.

**Métodos:**

Vinte e nove pacientes realizaram o teste de caminhada de 6 minutos em um ambulatório de reabilitação pulmonar, na cidade de Santo André (acima do nível do mar), em São Paulo, e na praia da Enseada no Guarujá (ao nível do mar), também em São Paulo. Destes, oito pacientes realizaram tanto na areia batida como no asfalto, para avaliar a existência de alguma alteração no desempenho durante o teste. Dados como frequência cardíaca, saturação de oxigênio, distância do teste e escala de Borg foram comparados.

**Resultados:**

Não encontramos diferença estatística em relação à saturação de oxigênio em repouso antes do início do teste de caminhada em Santo André 94,67±2,26% e ao nível do mar 95,56±2% (p=0,71). A saturação mínima obtida durante os testes foi de 87,27±6,54%, em Santo André, e de 89,10±5,41%, no Guarujá (p=0,098). Não houve diferença na distância percorrida e nos diferentes tipos de piso; a distância na areia foi de 387,75±5,02m e 375,00±40,88m no asfalto (p=0,654). Quanto à saturação durante a caminhada, a oximetria de pulso na areia foi de 95,12±1,80% e no asfalto foi de 96,87±1,64% (p=1,05), ou seja, o teste foi reprodutível em ambos os solos.

**Conclusão:**

A altitude não influenciou o desempenho do teste de caminhada realizado por pacientes com doença pulmonar moderada a grave, sendo reprodutível tanto acima quanto ao nível do mar, mesmo realizado em areia batida ou no asfalto.

## INTRODUÇÃO

Indivíduos com doença pulmonar obstrutiva crônica (DPOC) podem apresentar redução importante do desempenho físico devido a vários fatores, como hiperinsuflação dinâmica e o descondicionamento físico progressivo associado à inatividade,^(1)^ desencadeando limitações físicas e sociais, que acarretam na deterioração da qualidade de vida desses indivíduos.^(2)^

Em decorrência dessas alterações, a avaliação da capacidade física ou da capacidade de exercício em indivíduos com DPOC tornou-se alvo de muitos estudos. As causas da intolerância ao exercício físico em doentes com DPOC são tradicionalmente focadas nas limitações do sistema ventilatório (na troca gasosa),^(3,4)^ em anormalidades funcionais (como redução da força e resistência) e da bioenergética muscular (como a redução no consumo de oxigênio).^(5-7)^

Para avaliar a capacidade física de pacientes com DPOC, utiliza-se o teste de caminhada de 6 minutos (TC6’), o qual reflete de maneira mais acurada as limitações às Atividades de Vida Diária e se mostra mais sensível que testes em esteira.^(8)^ Os indivíduos com DPOC apresentam redução da tolerância ao exercício,^(9,10)^ associada à sensação de dispneia e de fadiga, e sofrem redução da capacidade funcional ao exercício, com a progressão da doença.^(9) ^Estudos realizados no Mar Morto mostraram que pacientes com DPOC apresentaram aumento da distância percorrida durante o TC6’, menor dispneia e melhora na capacidade funcional em menores altitudes.^(11-16)^

Os efeitos da altitude na fisiologia pulmonar decorrem basicamente da diminuição da pressão atmosférica. À medida que aumentamos a altitude a que estamos expostos, ocorre a diminuição da pressão atmosférica e, por consequência, a queda da pressão parcial de oxigênio no ambiente.^(17) ^No entanto, ainda não é bem estabelecida a interferência na variação do TC6’ em diferentes altitudes, como, por exemplo, entre a cidade de Santo André (São Paulo) e ao nível do mar.

A hipótese inicial do estudo era a de que os pacientes com doença pulmonar obstrutiva crônica de moderada a grave teriam melhor desempenho físico ao nível do mar durante o teste de caminhada de 6 minutos, independentemente do tipo de solo.

## OBJETIVOS

Avaliar a influência da altitude no teste de caminhada de 6 minutos em pacientes com doença pulmonar obstrutiva crônica moderada a grave, e o desempenho dos pacientes em diferentes solos, como na areia batida e no asfalto.

## MÉTODOS

Trata-se de um estudo retrospectivo, no qual foram analisados dados de 29 pacientes, sendo 19 mulheres. A média da idade dos pacientes foi 66,41±10,55 anos, com volume expiratório forçado no primeiro segundo (VEF1), em porcentagem, de 50,56±14,81% e índice de massa corporal (IMC) de 25,9±5,9. Eram portadores de DPOC moderada a grave, segundo GOLD,^(18)^ sendo 17 moderados e 12 graves (Tabela 1).

**Tabela 1:** Características demográficas da amostra

Características	n
Gênero	
Masculino	10
Feminino	19
Idade, anos	
Média	66,41
Desvio padrão	14,81
IMC, m/kg^2^	
Média	25,9
Desvio padrão	5,9
VEF1 pós% basal	
Média	50,56
Desvio padrão	14,81
DPOC	
Moderada	17
Grave	12

IMC: índice de massa corporal; VEF1: volume expiratório forçado no primeiro segundo; DPOC: doença pulmonar obstrutiva crônica.

Os pacientes realizaram o TC6’ no Ambulatório de Reabilitação Pulmonar da Faculdade de Medicina do ABC, no município de Santo André (acima do nível do mar 760m), em São Paulo, e na praia da Enseada, no Guarujá (ao nível do mar=0m), também no estado de São Paulo. O teste foi feito segundo as diretrizes da *American Thoracic Society* (ATS)^(19)^ em uma pista de 30m demarcada. O TC6’foi realizado durante uma atividade externa, que foi incluída no Programa de Reabilitação Pulmonar (PRP) como procedimento padrão do serviço no período de outubro de 2012 a abril de 2014. O projeto foi aprovado pelo Comitê de Ética em Pesquisa da Faculdade de Medicina do ABC (CAAE: 06661712.3.0000.0082) e todos pacientes assinaram Termo de Consentimento Livre e Esclarecido.

Foram incluídos pacientes com DPOC moderada à grave, com VEF1 entre 30% e <80%,^(18)^ clinicamente estáveis e em tratamento adequado para o grau da doença e em acompanhamento com pneumologista. Foram excluídos pacientes clinicamente instáveis e que tiveram exacerbações há pelo menos 3 meses, sem condições de caminhar (limitações osteomusculares ou neurológicas) e com doença cardíaca grave e/ou em uso de oxigênio.

Os pacientes realizaram o TC6’ 1 hora após a chegada à praia seguindo as diretrizes da ATS,^(19)^ não havendo um período de adaptação. Antes e depois dos testes, foram aferidos os parâmetros como pressão arterial sistólica (PAS) e diastólica (PAD), frequência cardíaca (FC), saturação de oxigênio (SaO_2_) e escala de Borg^(20)^ de dispneia e fadiga dos membros inferiores. No entanto, para segurança e melhor monitoramento dos sujeitos, foram aferidas a SaO_2_, FC e escala de Borg a cada 1 minuto até o fim do teste. Após completar o TC6’, a distância que cada indivíduo percorreu durante o teste foi registrada. Todos os parâmetros e as distâncias foram comparados entre o teste feito acima do nível do mar e ao nível do mar. Para aferir a SaO_2_ e a FC, foi utilizado oxímetro de pulso digital de dedo Onyx^®^, modelo 9500 (Nonin) (Medical, Inc. Minneapolis, MN, USA). Durante essa atividade, os pacientes foram acompanhados por pneumologistas, fisioterapeutas e uma auxiliar de enfermagem treinada para medidas de emergência.

Ao todo, 29 pacientes participaram do estudo. Dentre eles, oito pacientes realizaram o teste na praia em piso irregular (na areia batida) e no asfalto em 2 dias diferentes, com intuito de avaliar a desempenho deles em solos diferentes.

Os dados obtidos, da amostra total (n=29), durante os testes como SaO_2_ e a distância do TC6’, foram comparados e correlacionados. Foram realizados testes *t* pareado e de correlação entre eles com todas variáveis realizadas acima do nível do mar, ao nível do mar e em diferentes solos na praia (distância, SaO_2_ e escala de Borg). Para análise da escala de Borg, dispneia máxima e fadiga dos membros inferiores em Santo André e na praia foi realizado o teste de χ^2^. As únicas variáveis analisadas nos oito pacientes que fizeram os testes na areia e no asfalto foram a distância do TC6’ e a SaO_2_ durante os testes, uma vez que a amostra era pequena e a análise do χ^2^ não poderia ser realizada. Os resultados foram obtidos com média e desvio padrão. O teste de normalidade da amostra (mesmo sabendo que deveria ser feita com amostras maiores) foi realizado e optamos por usar teste *t *pareado, já que a mesma amostra foi comparada em diferentes situações. O cálculo do *r *foi feito pelo teste de Pearson.

## RESULTADOS

Não foram encontradas diferenças entre a SaO_2_ em repouso durante o TC6’ no Ambulatório em Santo André (94,76±2,26%) e na Praia da Enseada (95,54±2%; p=0,71). A SaO_2_ mínima obtida durante os testes foi de 87,28±6,54% em Santo André e 89,10±5,41% no Guarujá (p=0,098) (Tabela 2).

**Tabela 2:** Média das variáveis do teste de caminhada de 6 minutos nas diferentes altitudes

Altitude	Distância TC6’, m			SaO_2_ basal,%			SaO_2_ min,%		
	Média	Desvio padrão	Valor de p	Média	Desvio padrão	Valor de p	Média	Desvio padrão	Valor de p
Acima do nível do mar 760m	428,31	54,94	0,178	94,76	2,22	0,71	87,28	6,43	0,098
Ao nível do mar=0m	413,17	59,78		95,54	1,97		89,10	5,32	

SaO_2_: saturação de oxigênio; TC6’: teste de caminhada de 6 minutos.

Não houve diferença estatística nas distâncias durante o TC6’ realizado na praia e na altitude de Santo André. Na praia obteve-se uma média de 428,31±55,91m e em Santo André 413,17±60,8m (p=0,178), ou seja, os pacientes foram capazes de reproduzir o teste em altitudes diferentes (r=0,49; p=0,007) (Tabela 2) (Figura 1).

**Figura 1:** Comparação da distância percorrida no teste de caminhada de 6 minutos nas diferentes altitudes (n=29)

Uma amostra de oito pacientes realizou o TC6’ na praia e no asfalto e não houve diferença quanto à distância percorrida entre o tipo de piso (distância de 387,75±5,02m na areia e 375,00±40,88m no asfalto; p=0,654). Durante a caminhada na areia, obteve-se SaO_2_ de 95,54±1,97% e, no asfalto, de 96,88±1,54% (p=1,05), ou seja, o teste é reprodutível em ambos os solos (Tabela 3) (Figura 2).

**Tabela 3:** Média das variáveis do teste de caminhada de 6 minutos nos diferentes tipos de solo na praia

Tipos de solo	Distância TC6’ (m)			SaO_**2**_ basal (%)		
	Média	Desvio padrão	Valor de p	Média	Desvio padrão	Valor de p
Asfalto	375	38,24	0,654	96,88	1,54	1,05
Praia	387,75	5,02		95,54	1,97	

SaO_2_: saturação de oxigênio; TC6’: teste de caminhada de 6 minutos.

**Figura 2:** Comparação da distância percorrida no teste de caminhada de 6 minutos em diferentes pisos (n=8)

Para o Borg máximo de dispneia, não houve diferença de χ^2^ entre as duas situações (p=0,3), assim como não encontramos diferença significativa em relação ao Borg máximo de fadiga de membros inferiores (p=0,55), ou seja, a sensação de dispneia e fadiga de membros inferiores foi semelhante nas diferentes altitudes (Tabela 4).

**Tabela 4:** Resultados da escala de Borg(20) - dispneia e fadiga dos membros inferiores

Escala de Borg	Santo André (n=29)	Praia (n=29)	Asfalto (n=8)
Borg dispneia máxima	7	7	5
Borg fadiga máxima dos membros inferiores	5	5	5
Borg dispneia mínima	0	0	0
Borg fadiga mínima dos membros inferiores	0	0	0

## DISCUSSÃO

Pacientes com DPOC usualmente relatam que, ao nível do mar, a sensação de dispneia é menor do que na cidade de Santo André. Os testes em ambas as altitudes foram reprodutíveis, ou seja, eles tiveram o mesmo desempenho tanto ao nível do mar como a 760m de altitude (resultados semelhantes), independentemente do solo utilizado.

Os pacientes não apresentaram diferença na distância entre os TC6’ nas diferentes altitudes avaliadas e nem nos diferentes tipos de solo, porém estudo no qual foram analisados os gastos energético e mecânico de pessoas que correm e caminham na areia demonstrou que caminhar e correr requer 1,6-2,5 vezes mais esforço do que em superfície firme.^(21)^ Já estudo realizado no Mar Morto em pacientes com DPOC mostrou aumento na distância do TC6’ de 112m após permanecerem 1 semana em uma altitude de 417m abaixo do nível do mar e de 170m após 3 semanas. Uma semana após retornarem a Jerusalém, persistiram com um aumento da distância percorrida de 116m. Em nosso estudo, não obtivemos essa diferença, porém devemos considerar que o tempo que esses pacientes permaneceram ao nível do mar foi pequeno para que tivessem alguma alteração durante o TC6’.^(12) ^Além disso, a diferença de altitude foi menor (em metros) do que no trabalho citado. O TC6’ é um importante teste de avaliação da capacidade física de indivíduos com limitação funcional, nos quais a avaliação deve ser útil para quantificar a gravidade dessa limitação e os resultados do tratamento. Os indivíduos com DPOC apresentam redução da tolerância ao exercício,^(9)^ associada à sensação de dispneia e fadiga, e sofrem redução da capacidade funcional ao exercício, com a progressão da doença.^(10)^

Não encontramos diferença entre a SaO_2_ em repouso durante os testes de caminhada e na saturação mínima entre as diferentes altitudes. Em contrapartida, no estudo realizado no Mar Morto, os pacientes apresentaram, em Jerusalém, decréscimo de 88 para 84% de SaO_2 _ao final da caminhada. No Mar Morto, essa diminuição foi de 92 para 86%, após 1 semana, e de 93 para 83%, após 3 semanas.^(12)^ Ademais, outro estudo^(13)^ realizado no Mar Morto sugeriu que todos os pacientes se sentiam com menos dispneia e relatou melhora da capacidade funcional e menor necessidade de oxigênio. Possivelmente, o tempo de estadia em altitudes mais baixas influencie nos resultados obtidos e talvez seja necessário permanecer por pelo menos 1 semana em menores altitudes para avaliar as diferenças na capacidade física após esse período de adaptação.

Pacientes com DPOC apresentam dificuldade de sociabilização e de lazer, não acompanham familiares em eventos sociais, ou seja, isolam-se deixando cada vez mais de realizar tarefas e abandonando suas atividades.^(22)^ É importante enfatizar que quando uma pessoa é diagnosticada com DPOC, ela deve necessariamente modificar seu estilo de vida, buscando uma melhor qualidade de vida.^(23)^ Estimular o contato social, bem como promover o passeio desses pacientes na praia, faz parte do nosso PRP, com objetivo de promover melhora na qualidade de vida e na integração deles com a sociedade.

Algumas limitações podem ter influenciado nos resultados do presente estudo, como o pouco de tempo de permanência ao nível mar, a menor diferença entre as altitudes estudadas e o pequeno número da amostra. Como objeto de projeto futuro, seria interessante seguir esta linha de pesquisa e estudar pacientes que permaneçam pelo menos 1 semana ao nível do mar.

## CONCLUSÃO

A altitude não influenciou o desempenho dos pacientes com doença pulmonar obstrutiva crônica moderada a grave durante o teste de caminhada de 6 minutos. Os resultados das variáveis analisadas foram semelhantes, tanto ao nível do mar quanto na altitude da cidade de Santo André (760m).

A análise do desempenho relacionado aos diferentes pisos também não mostrou diferença significativa no teste de caminhada de 6 minutos realizado no asfalto ou na areia batida, sendo necessário realizá-lo em uma amostra maior.
